# Hyponatremia in Patients Admitted to Intensive Care Unit of a Tertiary Center: A Descriptive Cross-sectional Study

**DOI:** 10.31729/jnma.7123

**Published:** 2022-11-30

**Authors:** Suraj Parajuli, Sanjeeb Tiwari, Sanjay Kumar Gupta, Yogendra Man Shakya, Yagya Laxmi Shakya

**Affiliations:** 1 Department of Clinical Skills, Medical University of the Americas, Nevis, West Indies; 2 Department of General Practice and Emergency Medicine, Tribhuvan University Teaching Hospital, Maharajgunj, Kathmandu, Nepal

**Keywords:** *cross-sectional study*, *prevalence*, *sodium*

## Abstract

**Introduction:**

Hyponatremia is one of the common electrolyte abnormalities in intensive care unit settings. Hyponatremia may lead to an increased hospital stay, morbidity and mortality. Hyponatremia can occur due to a variety of iatrogenic as well as part of complex disease processes during hospital admission. The objective of this study was to find the prevalence of hyponatremia in patients admitted to the intensive care unit of a tertiary care centre.

**Methods:**

A descriptive cross-sectional study was conducted in the intensive care unit of a tertiary care centre for a duration of six months from 12 August 2015 to 11 January 2016. Ethical approval was taken from the Institutional Review Committee (Reference number: 124/6-11-E/072/073). Data was collected from hospital records. Patients with abnormal serum sodium levels after admission to the intensive care unit were included in the study. Hyponatremia was defined as a serum sodium level less than 135 mEq/L. Convenience sampling method was used. Point estimate and 95% Confidence Interval were calculated.

**Results:**

Among 102 patients, the prevalence of hyponatremia was found to be 21 (20.59%) (12.7428.44, 95% Confidence Interval).

**Conclusions:**

The prevalence of hyponatremia in patients admitted to the intensive care unit was higher than in other studies conducted in similar settings.

## INTRODUCTION

Hyponatremia is a common electrolyte disorder in hospitalised patients. Abnormal serum sodium concentrations are known to affect normal physiologic function and several studies have suggested hyponatremia to be associated with adverse outcomes. Critically ill patients admitted to the intensive care unit (ICU) are particularly vulnerable to hyponatremia due to the nature of the disease leading to ICU admission as well as iatrogenic intervention.^1,2^

Hyponatremia is one of the independent risk factors for mortality among medical and surgical patients.^3,4^ Early recognition of the symptoms, judicious use of life-saving fluids, and selection of medicines with knowledge of fluid status can be important predictors for decreasing morbidity, mortality, and the length of hospital stay of patients. However, the study of the prevalence of hyponatremia along with commonly associated symptomologies is rare in the ICU settings of Nepal.

This study is aimed to find out the prevalence of hyponatremia in patients admitted to the intensive care unit of a tertiary care centre.

## METHODS

A descriptive cross-sectional study was conducted in the ICU of Tribhuvan University Teaching Hospital (TUTH) for a duration of six months from 12 August 2015 to 11 January 2016. Ethical approval was obtained from the Institutional Review Board of the Institute of Medicine (Reference Number: 124/6-11-E/072/073). Informed written consent was taken from patients or patient's caretakers. Participants were enrolled after application of inclusion criteria of patients aged >18 years willing to participate with normal serum sodium measurement (135-145 mmol/L) at the time of ICU admission and who had ICU stay longer than 1 calendar day duration. Similarly, patients with preexisting dialysis dependence and patients who received renal replacement therapy on ICU admission day 1 were excluded from the study. Convenience sampling method was used. The sample size was calculated using the formula:


$$\begin{array}{ll} \text{n} & ={\text{Z}}^{2}\times \frac{\text{p}\times \text{q}}{{\text{e}}^{\text{2}}}\\ & =1.96^{2}\times \frac{0.177\times 0.823}{0.10^{2}}\\ & =56 \end{array}$$

Where,

n= minimum required sample size
Z= 1.96 at 95% Confidence Interval (CI)
p= prevalence of hyponatremia taken as 17.7%^4^
q= 1-p
e= margin of error, 10%

The calculated sample size was 56. By adding a 10% non-response rate, the total sample size was 102.

Demographic data of the sample population was collected, and physical examination and provisional diagnosis were noted. Serum sodium concentration during admission and any changes in levels during ICU stay was noted. Serum potassium, plasma glucose level, and length of ICU stay were also noted. Serum levels of sodium and potassium were determined by ion selective electrode method using a fully automated electrode analyzer EX-D S/N JE A1 5060050, Japan at TUTH Biochemistry laboratory. Hyponatremia was defined as a serum sodium level less than 135 mEq/L.^1^

Data were analysed using Microsoft Excel 2016 and IBM SPSS Statistics version 21.0. Point estimate and 95% CI were calculated.

## RESULTS

Out of 102 ICU admitted patients, 21 (20.59%) (12.7428.44, 95% CI) were found to have hyponatremia. The mean age of the study population was found to be 57.78±16.64 years. Hypertension was present in 7 (33.33%) of the patients (Table 1).

**Table 1:** Comorbidity among patients with hyponatremia (n= 21).

Comorbidity	n (%)
Diabetes mellitus	5 (23.81)
Hypertension	7 (33.33)

The average ICU stay in patients with hyponatremia was 6.62±1.9 days. The mean potassium value in patients with hyponatremia was 4.31±0.64 mEq/L.

**Figure 1:**
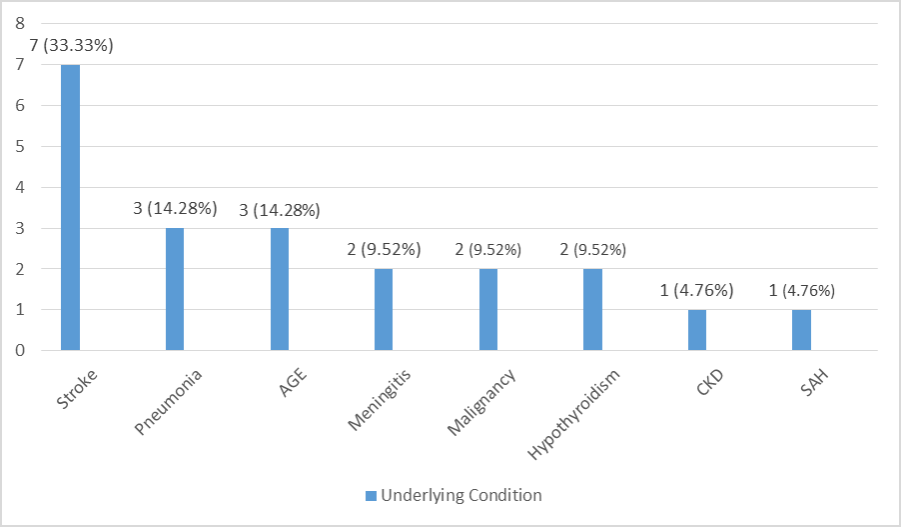
Underlying conditions among the patient with hyponatremia (n= 21).

Clinical features of hyponatremia were found in 9 (42.86%) patients, while 12 (57.14%) patients were asymptomatic. Out of the 9 (42.86%) patients who were symptomatic, confusion was the main feature occurring in 5 (55.56%) of the cases. Clinical symptoms such as comma, tremors, hallucinations, seizures and lethargy were also found. Among them, acute gastroenteritis (AGE) was present in 3 (14.28%), chronic kidney disease (CKD) in 1 (4.76%) and subacute haemorrhage (SAH) in 1 (4.76%) patients (Figure 1).

## DISCUSSION

Out of 102 patients admitted to ICU during the study period, 21 (20.59%) patients had hyponatremia. The prevalence of hyponatremia in this study was found to be higher than similar studies which revealed the prevalence of hyponatremia to be 17.7%.^1,2,4^ However, another study revealed the frequency of hyponatremia to be 29.83%.^5^ This variation may be due to an increase in sample size, multicentric study, duration of follow-up, and differences in the cohort of patients' symptoms during admission.

In this study, the mean age of the study population was found to be 57.78±16.64 years. The age group most affected by hyponatremia was seen in the 40-60 years group. The mean serum potassium level was found to be lower in hyponatremic patients than in hypernatremic patients which is in contrast with one study, which showed an inverse relationship between serum potassium level and serum sodium level.^5^

The average length of ICU stay for hyponatremia was 6.62±1.9 days. A different study done in similar settings revealed similar results.^4^ This relationship is likely to reflect multiple risk factors including increased illness severity in patients with long ICU stays, an increased exposure period to adverse events and clinician distractions as patients become chronically critically ill.^6,7^

Most of the patients with hyponatremia were asymptomatic (57.14%). The most common symptom seen in hyponatremia was confusion which is similar to other studies conducted in similar settings in India. However, asymptomatic cases were seen in only 20% of cases which is far lower than our study.^8^ These findings may be a result of a smaller sample size of our study. However, it could not be established whether the symptoms were solely due to low serum sodium levels or due to other primary causes, interventions/instrumentations in ICU following admission.

In this study, CNS pathologies were found to be in most cases 10 (47.61%) of the hyponatremic population. A study done in Africa concluded that head injury (30%), diabetes mellitus (22%), infection (13%), and gastrointestinal disturbances (13%) were the main underlying conditions in more than 75% of all patients with serum sodium derangements.^9^

The mortality rate in the hyponatremic group was found to be 14.29%. This finding is low in comparison to studies done in Dutch Medical ICU, Medical ICU in Austria, and medical ICU in France respectively.^10-12^ This may be pointed to a smaller sample size as well as the methodical approach of ICU personnel toward early detection and treatment of sodium disorders.

The results of this observational study cannot prove causality but can be used for generating a hypothesis. We speculate that avoidance of excessive fluids and appropriate treatment of hyponatremia may improve prognosis. This hypothesis should be clarified in a randomised prospective interventional trial using a treatment group and a control group.

There were some limitations to our study. Children and patients of age<18 years were excluded from the study population. This study was conducted in a single centre. Measurement of other parameters such as serum osmolality, urine osmolality, and types of medical interventions would have provided a much better insight into this study. This study could be confounded by various other factors that alter sodium balance which was not assessed.

## CONCLUSIONS

The prevalence of hyponatremia in patients admitted to the intensive care unit was higher than in other studies conducted in similar settings. This study shows hyponatremia occurs in ICU admitted patients. As in this study, the clinical features may not be apparent in many cases which makes serum sodium imbalances difficult to identify and manage accordingly. However, further multicentric studies are required to identify the prevalence of hyponatremia in ICU settings.
